# Acute respiratory failure and mechanical ventilation in cardiogenic shock complicating acute myocardial infarction in the USA, 2000–2014

**DOI:** 10.1186/s13613-019-0571-2

**Published:** 2019-08-28

**Authors:** Saraschandra Vallabhajosyula, Kianoush Kashani, Shannon M. Dunlay, Shashaank Vallabhajosyula, Saarwaani Vallabhajosyula, Pranathi R. Sundaragiri, Bernard J. Gersh, Allan S. Jaffe, Gregory W. Barsness

**Affiliations:** 1 Department of Cardiovascular Medicine, Mayo Clinic, 200 First Street SW, Rochester, MN 55905 USA; 2 Division of Pulmonary and Critical Care Medicine, Department of Medicine, Mayo Clinic, Rochester, MN USA; 3 Division of Nephrology and Hypertension, Department of Medicine, Mayo Clinic, Rochester, MN USA; 4 Department of Health Science Research, Robert D. and Patricia E. Kern Center for the Science of Health Care Delivery, Mayo Clinic, Rochester, MN USA; 5 Division of Hospital Internal Medicine, Department of Medicine, Mayo Clinic, Rochester, MN USA

**Keywords:** Cardiogenic shock, Acute myocardial infarction, Acute respiratory failure, Mechanical ventilation, Cardiac intensive care unit, Critical care cardiology, Outcomes research

## Abstract

**Background:**

There are limited epidemiological data on acute respiratory failure (ARF) in cardiogenic shock complicating acute myocardial infarction (AMI-CS). This study sought to evaluate the prevalence and outcomes of ARF in AMI-CS.

**Methods:**

This was a retrospective study of AMI-CS admissions during 2000–2014 from the National Inpatient Sample. Administrative codes for ARF and mechanical ventilation (MV) were used to define the cohorts of no ARF, ARF without MV and ARF with MV. Admissions with a secondary diagnosis of AMI and with chronic MV were excluded. Outcomes of interest included in-hospital mortality, temporal trends of ARF prevalence and resource utilization.

**Measurements and main results:**

During 2000–2014, 439,436 admissions for AMI-CS met the inclusion criteria. ARF and MV were noted in 57% and 43%, respectively. Admissions with non-ST-elevation AMI-CS, of non-White race and with non-private insurance received MV more frequently. Noninvasive ventilation and invasive MV increased from 0.4% and 39.2% (2000) to 3.6% and 46.4% (2014), respectively (*p *< 0.001). Coronary angiography and percutaneous coronary intervention were used less frequently in admissions receiving ARF with MV. Compared to admissions with no ARF, ARF without MV (adjusted odds ratio (aOR) 1.56 [95% confidence interval (CI) 1.53–1.59]; *p *< 0.001) and ARF with MV (aOR 2.50 [95% CI 2.47–2.54]; *p *< 0.001) were associated with higher in-hospital mortality. Admissions with ARF without MV had greater resource utilization and lesser discharges to home as compared to no ARF.

**Conclusions:**

In this contemporary AMI-CS cohort, the presence of ARF and MV use was noted in 57% and 43%, respectively, and was associated with higher in-hospital mortality.

## Introduction

Cardiogenic shock (CS) is seen in about 5–7% of patients with acute myocardial infarction (AMI) and is associated with high mortality and morbidity [1–4]. In patients with AMI-CS, use of early revascularization may reverse the hemodynamic insult limiting pump failure and subsequent hemodynamic compromise [5, 6]. However, patients with AMI-CS can present with varying degrees of hemodynamic compromise, fluid overload and end-organ hypoperfusion [6–9]. In addition, these patients typically have high filling pressures, biventricular failure and secondary pulmonary hypertension resulting in decreased gas exchange and increased work of breathing, contributing to acute respiratory failure (ARF) [8]. Prior literature has demonstrated that noninvasive ventilation (NIV) reduces respiratory distress and improves metabolic disturbances in acute cardiogenic pulmonary edema [10]. However, in patients with CS, NIV might not always be feasible due to the high metabolic demand from increased work of breathing, altered mental status resulting in poor synchrony, concomitant cardiac arrest and severity of pulmonary edema with poor diuretic response causing insufficient oxygenation, all of which require tracheal intubation and the use of invasive mechanical ventilation (IMV) [11].

There are limited large-scale epidemiological data on the use of mechanical ventilation (MV) in the USA [12, 13]. Prior epidemiological studies have looked at the role of MV in medical intensive care unit (ICU) and cardiac ICU populations [12, 13]. Recent data have noted increasing non-cardiac comorbidities in the cardiac ICU population, probably as a reflection of increasing severity of illness in this population [7, 13, 14]. Taking this background information into account, we sought to assess the epidemiology of ARF and MV in patients with AMI-CS in the USA. We hypothesized that during this 15-year study period, patients with AMI-CS have evolved into a more complex population with greater ARF and the use of MV, both NIV and IMV. We divided the population with AMI-CS into cohorts with no ARF, ARF without MV use and ARF with MV use.

## Materials and methods

### Study population, variables and outcomes

The National (Nationwide) Inpatient Sample (NIS) is the largest all-payer database of hospitalized inpatients in the USA and is a part of the Healthcare Cost and Utilization Project (HCUP), sponsored by the Agency for Healthcare Research and Quality [15]. During the study years, it contained data from about 1000 hospitals sampled to approximate a 20% sample of US community hospitals, defined by the American Hospital Association to be “all non-Federal, short-term, general and other specialty hospitals, excluding hospital units of institutions.” The strata use five hospital characteristics: ownership/control, bed size, teaching status, urban/rural location and US region. The sample of hospitals included each year is independent of the sample included in preceding years. Information regarding each discharge includes demographics, primary payer, hospital characteristics, principal diagnosis, up to 24 secondary diagnoses and procedural diagnoses.

Using the HCUP-NIS data from 2000 to 2014, a retrospective cohort study of admissions with AMI-CS was identified. Though the Agency for Healthcare Research and Quality has released the HCUP-NIS data till 2016, due to the change in coding practices from ICD-9CM to ICD-10CM in October 2015 we sought to restrict the data to 2014. The HCUP-NIS from 2015 and 2016 databases lacks the Clinical Classification System for ICD-9CM codes used in the study. Furthermore, the ICD-10CM codes lack extensive validation studies unlike the ICD-9CM codes and therefore need further evaluation prior to incorporation into temporal analyses [16, 17]. AMI in the primary procedure field was identified using International Classification of Diseases 9 Clinical Modification (ICD-9CM) codes for ST-elevation MI (STEMI) (ICD-9CM 410.1×–410.6×, 410.8×, 410.9×) and non-ST-elevation acute coronary syndrome (NSTEMI) (ICD-9CM 410.70–410.79) [18]. CS was identified using ICD-9CM code 785.51 and was defined as shock resulting from diminution of cardiac output in heart disease, shock resulting from primary failure of the heart in its pumping function, as in myocardial infarction, severe cardiomyopathy or mechanical obstruction or compression of the heart or shock resulting from the failure of the heart to maintain adequate output [19]. Validation studies have shown a specificity of 99.3%, a sensitivity of 59.8%, a positive predictive value of 78.8% and negative predictive value of 98.1% for the ICD-9CM code 785.51 to identify CS [19]. Admissions with CS due to non-AMI etiology and those without in-hospital mortality data were excluded. Using previous algorithms applied to the HCUP-NIS database, ARF was identified using the presence of any of the following ICD-9CM codes: (a) acute respiratory failure (ICD-9CM 518.81), (b) other pulmonary insufficiency including acute respiratory distress syndrome and acute respiratory insufficiency (ICD-9CM 518.82), (c) acute respiratory distress syndrome after shock or trauma (ICD-9CM 518.85), (d) respiratory distress not otherwise specified (ICD-9CM 786.09), (e) respiratory arrest (799.1) and (f) ventilator management (ICD-9CM 96.7, 96.70, 96.71 and 96.72) [7, 20–22]. Use of MV was identified using ICD-9CM codes for NIV (ICD-9CM 93.90) and IMV (ICD-9CM 96.7, 96.70, 96.71, 96.72) [12]. The ICD-9CM for NIV and IMV is 86% sensitive/92% specific and 86% sensitive/99.7% specific, respectively [23]. Since it is possible that NIV might have been used for other purposes outside of ARF (obstructive sleep apnea, sleep disorder breathing), we excluded admissions when NIV was used without a concomitant diagnosis of ARF. Demographic and hospital characteristics associated with each discharge were identified from the HCUP-NIS database. Prior validated methodology was used to define acute organ dysfunction, cardiac and non-cardiac procedures [7, 22, 24–29]. The Deyo’s modification of the Charlson comorbidity index was used to identify the burden of comorbid diseases (Additional file 1: Table S1) [30]. The hospital day on which the procedure was performed was used to identify the use of NIV before or after IMV in admissions that received both modalities.

The primary outcome was the in-hospital mortality in AMI-CS stratified into cohorts with no ARF, ARF without MV and ARF with MV. Secondary outcomes included the prevalence, temporal trends of ARF, length of stay, costs, use of do-not-resuscitate status and discharge disposition in admissions with ARF with/without MV.

### Statistical analysis

As recommended by HCUP-NIS, survey procedures using discharge weights provided with HCUP-NIS database were used to generate national estimates. As recommended by HCUP-NIS, survey procedures using discharge weights provided with HCUP-NIS database were used to generate national estimates. Using the trend weights provided by the HCUP-NIS, samples from 2000 to 2011 were re-weighted to adjust for the 2012 HCUP-NIS re-design [31]. In 2012, the HCUP-NIS was re-designed to sample 20% of the national patient-level sample as compared to 2000–2011 wherein it sampled 100% of the discharges from 20% of the hospitals [31]. Using trend weights available on the HCUP-NIS database, samples from 2000 to 2011 were retroactively re-weighted. The new sampling strategy is expected to result in more precise estimates than the previous HCUP-NIS design by reducing sampling error [15]. This methodology has been used by multiple prior studies spanning across year 2012 from the HCUP-NIS [7, 22, 24–29]. One-way analysis of variance (ANOVA) and *t*-tests were used to compare categorical and continuous variables, respectively. The inherent restrictions of the HCUP-NIS database related to research design, data interpretation and data analysis were reviewed and addressed [31]. Univariate analysis for trends of ARF and in-hospital mortality stratified by ARF with/without MV was represented as odds ratio (OR) with 95% confidence interval (CI). For the adjusted analysis, a multivariable logistic regression analysis including age, sex, race, admission year, primary payer status, socioeconomic stratum, hospital characteristics, comorbidities, acute organ dysfunction, severe sepsis, cardiac arrest, cardiac procedures, mechanical circulatory support and hemodialysis was performed for in-hospital mortality. For the multivariable modeling, purposeful selection of statistically (*p *< 0.20) and a priori selected clinically relevant variables was conducted. Additionally, we performed a propensity-matched analysis for demographics, comorbidities, hospital characteristics, acute organ failure and acute care interventions between the two cohorts. For the propensity matching, all variables except race had < 1% missing variables. For the race category, missing variables were imputed using random sampling from the respective covariate distributions. Using 1:1 nearest-neighbor matching, 9240 matching pairs (18,480 individual admissions) were developed for further use. The propensity-matched sample had standardized differences < 10% for all baseline characteristics. The McNemar *χ*^2^ test and paired sample *t*-tests were used to compare categorical and continuous variables, respectively, in the propensity-matched sample. Two-tailed *p *< 0.05 was considered statistically significant. All statistical analyses were performed using SPSS version 25.0 (IBM Corp, Armonk, NY).

## Results

**Fig. 1:**
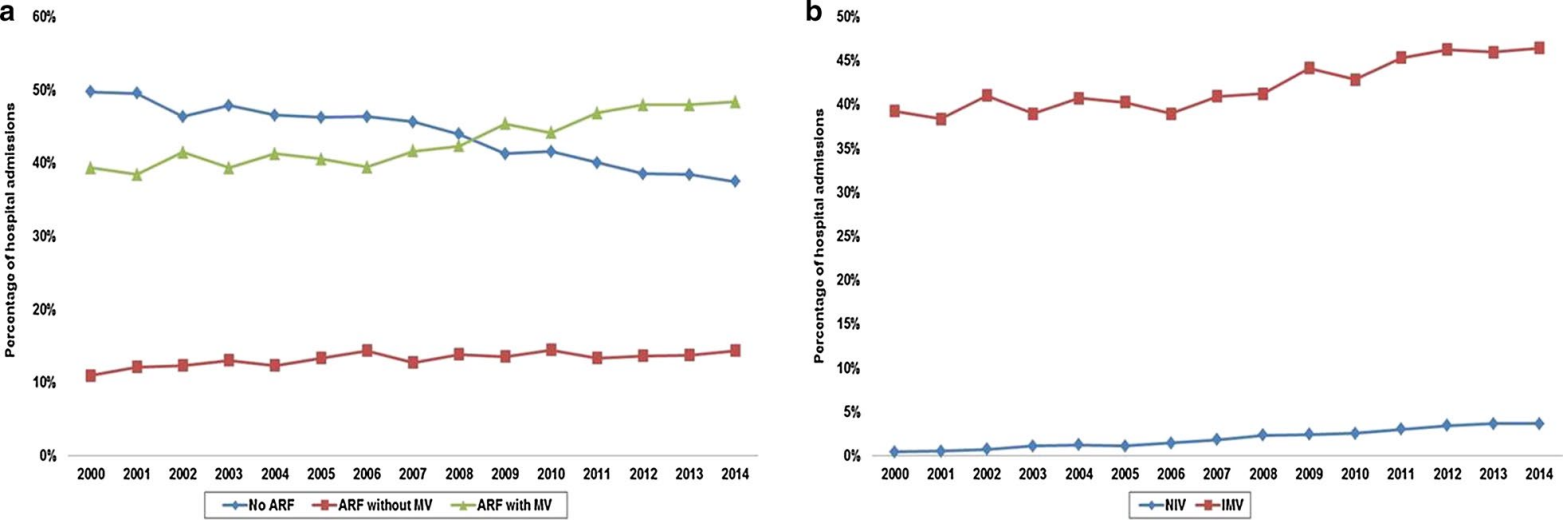
Prevalence of ARF and MV in AMI-CS. **a** 15-year trends in the incidence of no ARF, ARF without MV and ARF with MV in AMI-CS; **b** 15-year trends in NIV and IMV use in AMI-CS with ARF; all *p *< 0.001 for trend. *AMI* acute myocardial infarction, *ARF* acute respiratory failure, *CS* cardiogenic shock, *IMV* invasive mechanical ventilation, *MV* mechanical ventilation, *NIV* noninvasive ventilation

**Fig. 2:**
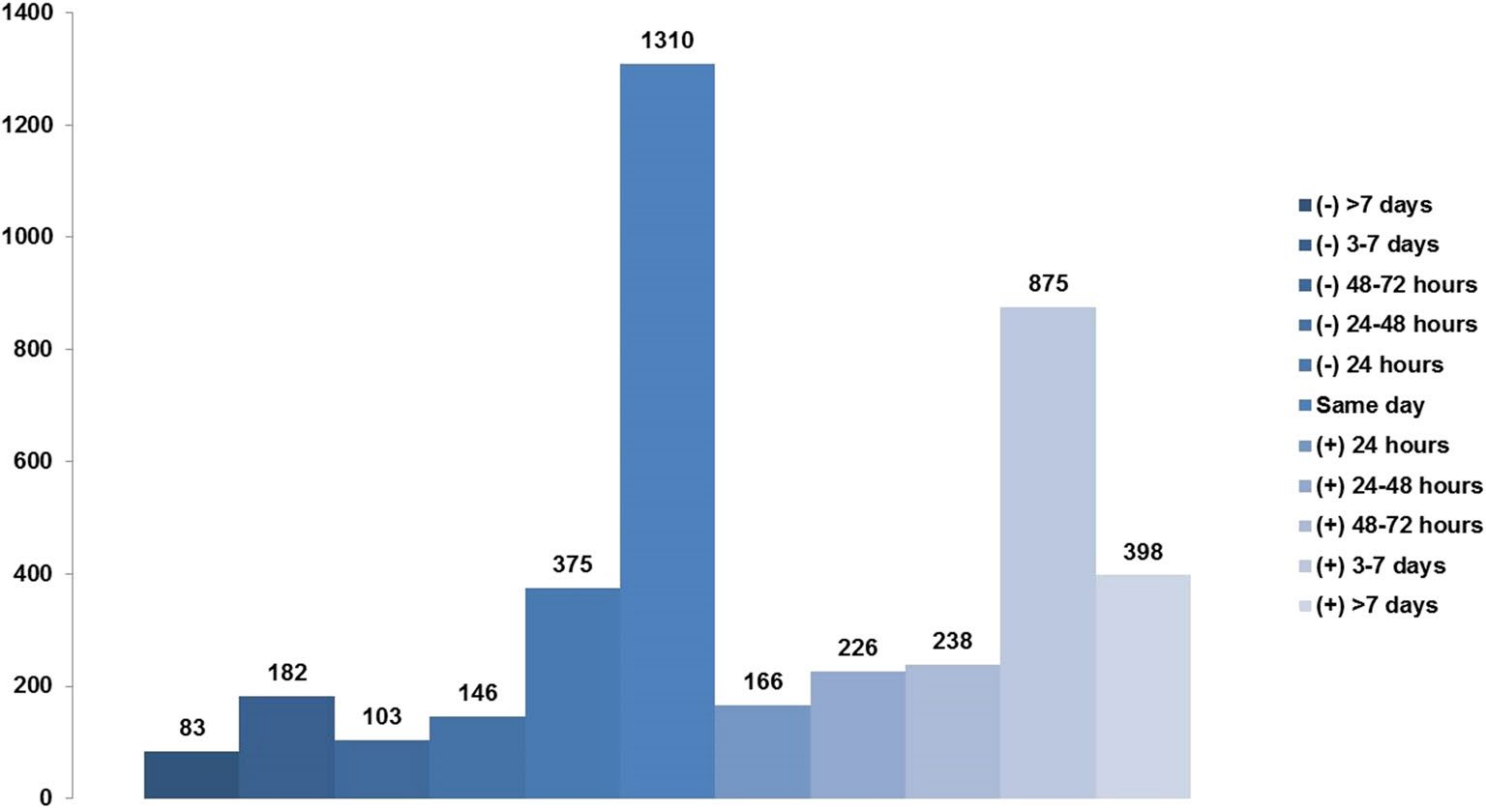
Timing of NIV with relation to IMV (*N* = 3866). Negative values denote NIV use before IMV and positive values denote NIV use after IMV. *IMV* invasive mechanical ventilation, *NIV* noninvasive ventilation

There were an estimated number of 444,253 admissions for AMI-CS between January 1, 2000, and December 31, 2014, that met criteria for a primary diagnosis of STEMI or NSTEMI. An estimated number of 4817 (1.1%) admissions received NIV without a concomitant diagnosis of ARF and were excluded. In the final cohort of 439,436 admissions, ARF was noted in 247,898 (56.5%) with use of MV in 189,848 (43.2%). In these 189,848 admissions, NIV was used in 8895 (4.7%), IMV in 185,589 (97.8%) and both in 4636 (2.4%). Baseline characteristics of the cohorts with no ARF, ARF without MV and ARF with MV are summarized in Table 1. MV was used more frequently among admissions with NSTEMI-CS, of non-White race and with non-private insurance and to urban teaching hospitals. Over the 15-year study period, there was a steady decline in AMI-CS with no ARF with a concomitant increase in AMI-CS with ARF needing MV (Fig. 1a, b). Epidemiological trends of ARF and MV stratified by demographic and hospital characteristics are presented in Additional file 2: Figure S1 and Additional file 3: Figure S2. The timing of NIV with respect to IMV was available in 3866/4636 (83.4%) admissions (Fig. 2). Nearly one-third admissions received NIV and IMV on the same day (Fig. 2). Admissions with ARF needing MV had higher rates of concomitant cardiac arrest, acute kidney injury and invasive hemodynamic assessment (Table 1). Coronary angiography and percutaneous coronary intervention were used less frequently in ARF with MV (Table 1). Admissions with ARF without MV received mechanical circulatory support more frequently compared to those with no ARF; however, those with ARF with MV received it less frequently compared to ARF without MV.

**Table 1:** Baseline and hospital characteristics of AMI-CS with and without ARF

Characteristic	No ARF (*N* = 191,538)	ARF without MV (*N* = 58,050)	ARF with MV (*N* = 189,848)	*p*
AMI type				
STEMI	69.8	69.1	66.5	< 0.001
NSTEMI	30.2	30.9	33.5	< 0.001
Age (years)	69.5 ± 13.6	68.7 ± 12.8	69.1 ± 13.1	< 0.001
Female sex	40.3	38.5	38.2	< 0.001
Race				
White	63.1	60.0	63.9	< 0.001
Non-White	36.9	40.0	36.1	
Weekend admission	26.3	27.4	27.4	< 0.001
Primary payer				
Medicare	61.5	60.3	61.7	< 0.001
Medicaid	5.5	6.3	7.2	
Others	33.0	33.4	31.1	
Quartile of median household income for zip code				
0–25th	22.8	24.7	23.2	< 0.001
26th–50th	26.8	27.0	26.2	
51st–75th	25.2	24.3	25.0	
75th–100th	25.2	24.0	25.6	
Hospital teaching status and location				
Rural	9.6	6.3	5.7	< 0.001
Urban non-teaching	41.4	40.1	40.2	
Urban teaching	49.0	53.6	54.2	
Hospital bed size				
Small	8.3	7.4	7.3	< 0.001
Medium	22.3	21.6	22.1	
Large	69.4	71.0	70.6	
Hospital region				
Northeast	18.3	15.2	19.6	< 0.001
Midwest	23.3	23.1	22.5	
South	38.8	44.9	35.9	
West	19.5	16.8	22.0	
Charlson comorbidity index				
0–3	27.0	23.6	22.0	< 0.001
4–6	53.3	57.4	57.2	
≥ 7	19.7	19.0	20.8	
Comorbidities				
Hypertension	51.5	42.7	51.0	< 0.001
Hyperlipidemia	35.9	25.7	29.1	< 0.001
Diabetes mellitus	4.0	4.5	4.7	< 0.001
Cancer	7.5	5.1	6.6	< 0.001
CKD	12.1	13.5	14.5	< 0.001
Heart failure	49.6	62.6	59.0	< 0.001
Cardiac arrest	8.2	18.6	28.0	< 0.001
Acute kidney injury	24.7	40.9	43.7	< 0.001
Coronary angiography	68.6	75.7	64.9	< 0.001
Percutaneous coronary intervention	48.8	56.4	43.4	< 0.001
Invasive hemodynamic assessment^a^	15.6	22.2	23.7	< 0.001
Severe sepsis	2.6	6.7	9.3	< 0.001
Cardiac surgery				
CABG	19.3	21.7	13.9	< 0.001
Valve surgery	1.6	3.2	1.9	< 0.001
MCS				
Total	41.5	56.7	45.5	< 0.001
IABP	40.6	54.6	44.0	< 0.001
pMCS	0.8	2.2	1.7	< 0.001
npMCS	0.4	0.9	0.5	< 0.001
ECMO	0.3	1.2	0.6	< 0.001
Hemodialysis	1.5	2.8	5.7	< 0.001

Represented as percentage or mean ± standard deviation

*AMI* acute myocardial infarction, *ARF* acute respiratory failure, *CABG* coronary artery bypass grafting, *CKD* chronic kidney disease, *CS* cardiogenic shock, *ECMO* extracorporeal membrane oxygenation, *IABP* intra-aortic balloon pump, *MCS* mechanical circulatory support, *MV* mechanical ventilation, *npMCS* non-percutaneous mechanical circulatory support, *NSTEMI* non-ST-elevation myocardial infarction, *pMCS* percutaneous mechanical circulatory support, *STEMI* ST-elevation myocardial infarction

^a^Right heart catheterization or pulmonary artery catheterization; all comparisons made using one-way analysis of variance

**Fig. 3:**
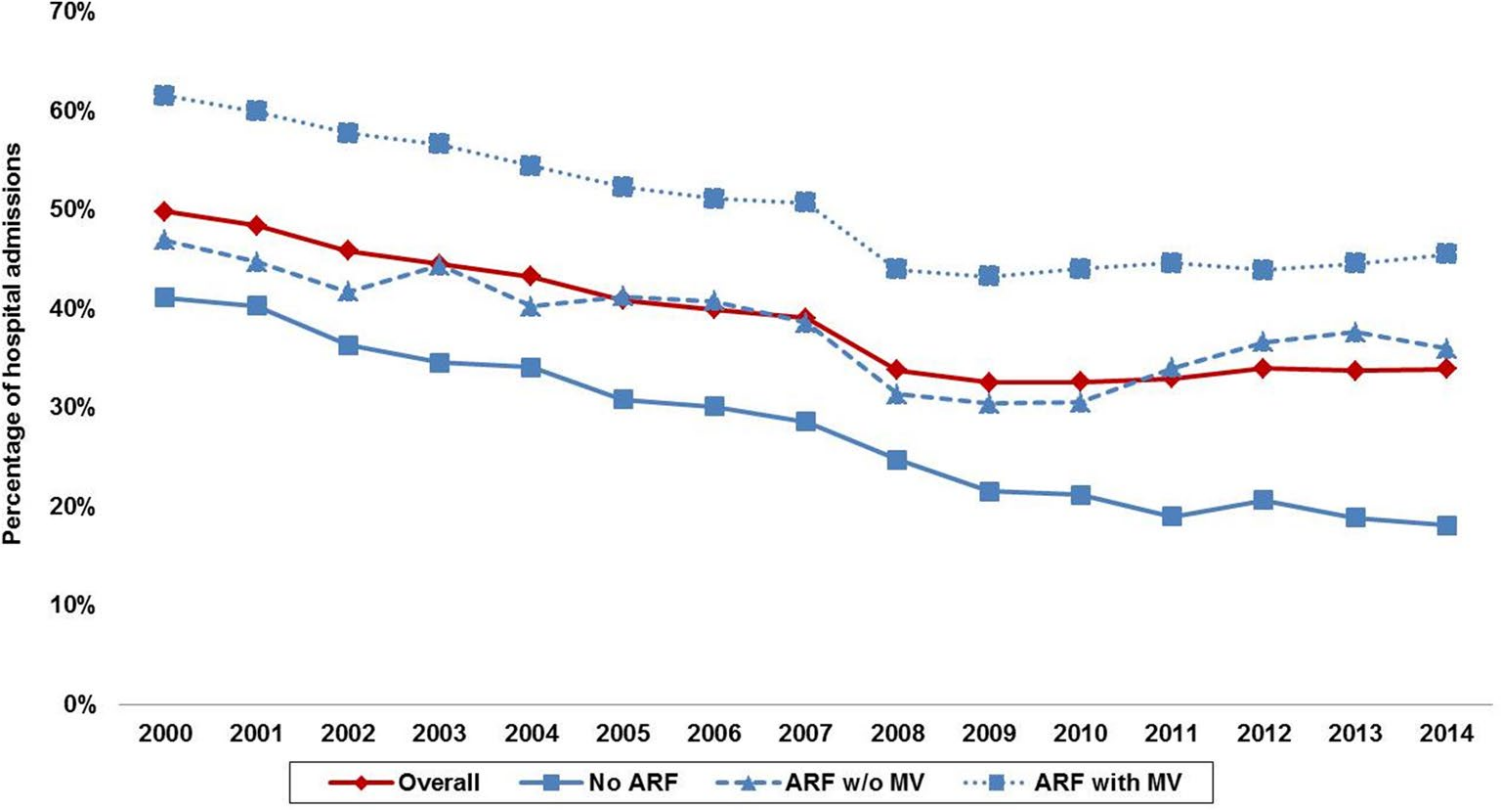
Trends of in-hospital mortality in AMI-CS stratified by ARF and MV. All *p *< 0.001. *AMI* acute myocardial infarction, *ARF* acute respiratory failure, *CS* cardiogenic shock, *MV* mechanical ventilation

Compared to those with no ARF, ARF without MV (37.8% vs. 28.1%; OR 1.56 [95% CI 1.53–1.59]; *p *< 0.001) and ARF with MV (49.4% vs. 28.1%; OR 2.50 [95% CI 2.47–2.54]; *p *< 0.001) were associated with higher in-hospital mortality. In-hospital mortality for the overall population and the ARF cohorts demonstrated a steady decline over the study period (Fig. 3). Admissions with ARF without MV had a longer length of stay, higher hospital costs and lesser discharges to home as compared to those without ARF (Table 2). Admissions with ARF with MV had lower hospital costs and lengths of stay compared to ARF without MV but had greater use of do-not-resuscitate status (5.6% vs. 4.5%; *p *< 0.001). In a multivariate regression analysis, compared to the cohort with no ARF, ARF without MV (OR 1.68 [95% CI 1.64–1.72]; *p *< 0.001) and ARF with MV (OR 2.21 [95% CI 2.17–2.25]; *p *< 0.001) were independently associated with higher in-hospital mortality in AMI-CS (Additional file 1: Table S2) (c-index 0.80; Hosmer and Lemeshow test for goodness of fit: *χ*^2^ 1019; *p *< 0.001). In a propensity-matched analysis (Additional file 1: Table S3), compared to those without MV, the cohort receiving MV (38% vs. 44.2%; OR 1.29 [95% CI 1.22–1.37]; *p *< 0.001) continued to demonstrate higher in-hospital mortality.

**Table 2:** Clinical outcomes of AMI-CS with and without ARF

Characteristic	No ARF (*N* = 191,538)	ARF without MV (*N* = 58,050)	ARF with MV (*N* = 189,848)	*p*
In-hospital mortality	28.1	37.8	49.4	< 0.001
Median length of stay (days)	8.1 ± 8.9	12.4 ± 12.6	11.5 ± 13.4	< 0.001
Median hospitalization costs (×1000 USD)	95 ± 115	159 ± 181	148 ± 176	< 0.001
Do-not-resuscitate status	3.0	4.5	5.6	< 0.001
Discharge disposition				
Home	37.3	23.2	15.7	< 0.001
Transfer	6.9	5.6	7.6	
SNF	15.7	21.9	18.6	
Home with HHC	11.7	11.1	8.3	
AMA	0.3	0.2	0.3	

Represented as percentage or mean ± standard deviation; all comparisons made using one-way analysis of variance

*AMA* against medical advice, *AMI* acute myocardial infarction, *ARF* acute respiratory failure, *CS* cardiogenic shock, *HHC* home health care, *MV* mechanical ventilation, *SNF* skilled nursing facility, *USD* US dollars

## Discussion

In this nationally representative population of AMI-CS, we noted a steady increase in the proportion of admissions with ARF and greater use of NIV and IMV between 2000 and 2014. Acute respiratory failure was seen more commonly in admissions with NSTEMI-CS, of non-White race and of male sex. The population with ARF with MV was less likely to receive coronary angiography and percutaneous coronary intervention. Acute respiratory failure without and with MV was associated with a 1.7- and 2.2-fold higher in-hospital mortality independent of baseline characteristics, the severity of illness and organ support. Similar findings were noted in the propensity-matched cohort.

### Epidemiology of acute respiratory failure in AMI-CS

Acute respiratory failure requiring MV continues to be a leading reason for admission to the ICU. In unselected critically ill patients, Mehta et al. [12] noted a steady increase in the use of IMV in the USA between 1993 and 2009. In this study, the subgroup with heart failure (without CS) was noted to have a relatively steady usage of IMV during the study period. Using a registry of 219 patients, Hongisto et al. [11] described the use of NIV and IMV in unselected CS. They noted a 12% overall incidence of NIV use and 63% IMV use during the 2-year study period. In contrast to these studies, our findings demonstrate an increasing incidence of ARF requiring MV. Furthermore, the use of NIV was noted in only 4.7% of our study population as compared to 12% in the CardShock trial [11]. In our study, the use of MV was noted in 43.2% of the population, which was significantly lower than the CardShock and IABP-SHOCK II (Intra-aortic Balloon Pump in Cardiogenic Shock II) cohorts [11, 32]. This can possibly be explained by the vast heterogeneity in the definition of CS employed in real-world registry data as compared to trial definitions. Additionally, differences in patient acuity and treatment between the USA and European populations may contribute to these differences. Our findings are consistent with data from other epidemiological studies that show greater use of IMV in male patients, non-White race and lower socioeconomic status [12]. In a population of 3.2 million non-cardiogenic ARF, Cooke et al. noted consistently higher rates of ARF in non-White patients. The reasons for these disparities are incompletely understood and may be due to decreased access to health care, late presentation, differences in cultural and religious beliefs and treatment preferences. Further quantitative research investigating these racial disparities is warranted.

Expectedly, we noted ARF to be associated with higher occurrence of end-organ failure and cardiac arrest. In patients with CS, biventricular dysfunction may result in complex hemodynamics in the setting of positive pressure MV [33, 34]. Since the HCUP-NIS database does not record hemodynamic or echocardiographic data, we could not discern bi- from single-ventricular failure among participants. Furthermore, patients with AMI-CS frequently develop acute metabolic acidosis and vasoplegic shock [35], so it is conceivable that they develop a capillary leak syndrome or acute respiratory distress syndrome from concomitant sepsis resulting in worsening ARF. The increasing rates of MV in this study is consistent with prior studies that note greater acuity of patients being admitted to cardiac ICUs [13, 14]. This has significant implications on the models of care and staffing in modern cardiac ICUs, which include but are not limited to, dual-trained cardiac intensivists, co-management of patients by cardiologists and intensivists and development of specialized nursing care covering aspects unique to both cardiac and medical ICU populations [36].

### Mortality with acute respiratory failure in AMI-CS

Multiorgan failure has been recognized as a significant contributor to morbidity and mortality in unselected medical and cardiac ICU patients [14, 37]. Recent AMI-CS prognostic scores have sought to incorporate measures of end-organ hypoperfusion into the risk stratification of these patients [7, 38, 39]. Consistent with these data, our study highlighted the incremental in-hospital mortality in admissions with ARF and with MV use. It is important to note that patients with ARF and MV received lesser mechanical circulatory support and had higher mortality and lesser utilization. Taken in aggregate, these data may suggest that use of MV was a marker of higher illness severity and therefore these patients died earlier during their hospital course. Our data can be readily compared to the subgroup of patients with CS (*n* = 600) enrolled in the Acute Heart Failure Database (AHEAD) registry [40]. In this study of unselected CS patients, the use of NIV, IMV or both was associated with 69%, 72% and 68% mortality, respectively, which was significantly higher than patients not receiving MV (i.e., 40%) [40]. The mortality rate for the cohort with ARF and MV was only 40% in our study, which is similar to the data from the CardShock registry [11]. These observed differences are likely due to multiple factors: (a) the CardShock population had a higher proportion of AMI-CS as compared to the AHEAD registry and patients who develop post-cardiotomy CS appear to be systematically different from the AMI-CS [8]; (b) the AHEAD registry represented a referral population to centers of excellence in Europe, which cannot be generalized to our study that is more representative of the national practice; and (c) there were significant differences in the use of specific vasoactive medications (such as levosimendan) that are not available in the USA, thereby preventing direct comparisons. In 219 patients with AMI-CS, Hongisto et al. did not note the MV strategy (NIV or IMV) to impact clinical outcomes in their population. Further dedicated studies are needed to understand the role, indications and contraindications to NIV in CS [8].

### Limitations

This study has several limitations, despite the HCUP-NIS database’s attempts to mitigate potential errors by using internal and external quality control measures. The ICD-9CM codes for AMI and CS have been previously validated that reduces the inherent errors in the study [18, 19]. Important factors such as the timing of ARF, the presence of ARF at admission and treatment-limiting decisions of organ support could not be reliably identified in this database. It is possible that there may be a hesitancy to intubate older or complicated patients that is reflected in the lower use of MV in this population. Importantly, change in respiratory function during the hospital stay (improvement or decline) could not be reliably assessed in all admissions, though an indirect assessment was available in admissions with a listed procedure day for NIV and IMV use. It is possible that despite best attempts at controlling for confounders by multivariate analysis, the use of MV is a marker of greater illness severity due to residual confounding. Echocardiographic data, mechanical ventilation data, sedation and paralysis, vasoactive medications and hemodynamic parameters were unavailable in this database. It is possible that sensitive definitions of ARF and the use of MV at lower thresholds of acuity may contribute to the increase in the prevalence of ARF and MV. However, the concomitant rise in other organ failure refutes this possibility. Despite these limitations, this study addresses an important knowledge gap highlighting the epidemiology of ARF and the use of MV in AMI-CS in a contemporary 15-year period.

## Conclusions

In this study of 439,436 admissions with AMI-CS, ARF affected nearly 57% of the total cohort with a significant increase in the use of MV over time. Acute respiratory failure with or without MV use was independently associated with higher in-hospital mortality. Further research is needed to understand the delicate cardiopulmonary interactions in AMI-CS with an emphasis on ways to prevent and limit the severity of concomitant ARF.

## Supplementary information

**Additional file 1: Table S1.** Administrative codes used for identification of diagnoses and procedures. **Table S2.** Multivariable regression for in-hospital mortality in AMI-CS. **Table S3.** Baseline characteristics of propensity-matched cohorts of AMI-CS.

**Additional file 2: Figure S1.** Trends of ARF and MV in AMI-CS stratified by demographic characteristics. Fifteen-year trends in acute respiratory failure (solid line) and mechanical ventilation (dashed line) in admission stratified by age groups (2A), race (2B), sex (2C) and Charlson comorbidity index groups (2D); all *p *< 0.001. AMI: acute myocardial infarction; ARF: acute respiratory failure; CS: cardiogenic shock; MV: mechanical ventilation.

**Additional file 3: Figure S2.** Trends of ARF and MV in AMI-CS stratified by hospital characteristics. Fifteen-year trends in acute respiratory failure (solid line) and mechanical ventilation (dashed line) in admission stratified by hospital location and teaching status (3A), hospital bed size (3B) and hospital region (3C); all *p*<0.001. AMI: acute myocardial infarction; ARF: acute respiratory failure; CS: cardiogenic shock; MV: mechanical ventilation.

## Data Availability

The datasets used and/or analyzed during the current study are publicly available with the Agency for Healthcare Research and Quality.

## Abbreviations

AMI: acute myocardial infarction
ARF: acute respiratory failure
CI: confidence interval
CS: cardiogenic shock
HCUP: Healthcare Cost and Utilization Project
ICD-9CM: International Classification of Diseases-9 Clinical Modification
ICU: intensive care unit
IMV: invasive mechanical ventilation
MV: mechanical ventilation
NIS: National Inpatient Sample
NIV: noninvasive ventilation
NSTEMI: non-ST-elevation myocardial infarction
OR: odds ratio
STEMI: ST-elevation myocardial infarction
