# Supplementary material for: Acute respiratory failure and mechanical ventilation in cardiogenic shock complicating acute myocardial infarction in the USA, 2000–2014
Source: Ann Intensive Care. 2019 Aug 28;9:96. doi: 10.1186/s13613-019-0571-2 (PMC6713772; doi:10.1186/s13613-019-0571-2)
Supplement: Supplementary file 1 — Additional file 1: Table S1. Administrative codes used for identification of diagnoses and procedures. Table S2. Multivariable regression for in-hospital mortality in AMI-CS. Table S3. Baseline characteristics of propensity-matched cohorts of AMI-CS. [file 13613_2019_571_MOESM1_ESM.docx]

**Table S1. Administrative codes used for identification of diagnoses and procedures**

| **Comorbidity** | **ICD-9CM Codes** |
| --- | --- |
| Cardiac arrest | 427.5 |
| Acute kidney injury | 584, 584.5, 584.6, 584.7, 584.8, 584.9 |
| Coronary angiography | 36.06, 37.22, 37.23, 88.53-88.56 |
| Percutaneous coronary intervention | 00.66, 36.01, 36.02, 36.05, 36.07, 88.57 |
| Invasive hemodynamic assessment | 37.21, 37.23, 204 (Clinical Classification Software) |
| Intra-aortic balloon pump | 37.61 |
| Percutaneous MCS | 37.68 |
| Non-percutaneous MCS | 37.60, 37.62, 37.65 |
| ECMO | 39.65 |
| Invasive mechanical ventilation | 96.7, 96.70, 96.71, 96.72 |
| Non-invasive ventilation | 93.90 |
| Hemodialysis | 39.95 |
| Hepatic failure | 570.0, 572.2, 573.3, 573.4 |
| Hematologic failure | 286.6-286.9, 287.4, 287.5 |
| Neurological failure | 293, 293.0, 293.1, 293.8, 293.81-293.84, 293.89, 293.9, 348.1, 348.3, 348.30, 348.81, 348.39, 780.01, 780.09, 89.14 |

**Abbreviations:** ECMO: extra-corporeal membrane oxygenation; ICD-9CM: International Classification of Diseases, 9.0 Clinical Modification; MCS: mechanical circulatory support

**Table S2. Multivariable regression for in-hospital mortality in AMI-CS**

| **Total cohort**  **(N = 439,436)** | | **Odds ratio** | **95% confidence interval** | | ***P*** |
| --- | --- | --- | --- | --- | --- |
|  |  |  | **Lower Limit** | **Upper Limit** |  |
| **Acute Respiratory Failure** | **No ARF** | Reference category | | | |
|  | **ARF without MV** | 1.68 | 1.64 | 1.72 | <0.001 |
|  | **ARF with MV** | 2.21 | 2.17 | 2.25 | <0.001 |
| **Age groups (years)** | **19-49** | Reference category | | | |
|  | **50-59** | 1.28 | 1.23 | 1.33 | <0.001 |
|  | **60-69** | 1.84 | 1.76 | 1.92 | <0.001 |
|  | **70-79** | 2.92 | 2.79 | 3.06 | <0.001 |
|  | **≥80** | 4.79 | 4.57 | 5.02 | <0.001 |
| **Female sex** | | 1.13 | 1.11 | 1.15 | <0.001 |
| **Race** | **White** | Reference category | | | |
|  | **Black** | 0.99 | 0.96 | 1.03 | 0.68 |
|  | **Hispanic** | 1.04 | 1.01 | 1.07 | 0.01 |
|  | **Asian** | 0.98 | 0.94 | 1.03 | 0.48 |
|  | **Native American** | 1.09 | 0.98 | 1.21 | 0.11 |
|  | **Others** | 0.99 | 0.95 | 1.03 | 0.66 |
| **Year of admission** | **2000** | Reference category | | | |
|  | **2001** | 1.01 | 0.97 | 1.05 | 0.67 |
|  | **2002** | 0.88 | 0.85 | 0.92 | <0.001 |
|  | **2003** | 0.79 | 0.75 | 0.82 | <0.001 |
|  | **2004** | 0.71 | 0.68 | 0.74 | <0.001 |
|  | **2005** | 0.69 | 0.66 | 0.72 | <0.001 |
|  | **2006** | 0.67 | 0.64 | 0.70 | <0.001 |
|  | **2007** | 0.63 | 0.60 | 0.65 | <0.001 |
|  | **2008** | 0.47 | 0.45 | 0.49 | <0.001 |
|  | **2009** | 0.42 | 0.40 | 0.44 | <0.001 |
|  | **2010** | 0.43 | 0.41 | 0.45 | <0.001 |
|  | **2011** | 0.42 | 0.40 | 0.44 | <0.001 |
|  | **2012** | 0.41 | 0.40 | 0.43 | <0.001 |
|  | **2013** | 0.41 | 0.40 | 0.43 | <0.001 |
|  | **2014** | 0.40 | 0.39 | 0.42 | <0.001 |
| **Primary payer** | **Medicare** | Reference category | | | |
|  | **Medicaid** | 0.94 | 0.91 | 0.98 | 0.001 |
|  | **Private** | 0.76 | 0.75 | 0.78 | <0.001 |
|  | **Uninsured** | 1.32 | 1.27 | 1.38 | <0.001 |
|  | **No Charge** | 0.85 | 0.76 | 0.96 | 0.009 |
|  | **Others** | 0.86 | 0.81 | 0.90 | <0.001 |
| **Quartile of median household**  **income for zip code** | **0-25^th^** | Reference category | | | |
|  | **26^th^-50^th^** | 0.93 | 0.91 | 0.95 | <0.001 |
|  | **51^st^-75^th^** | 0.90 | 0.88 | 0.92 | <0.001 |
|  | **75^th^-100^th^** | 0.87 | 0.85 | 0.89 | <0.001 |
| **Hospital teaching**  **status and location** | **Rural** | Reference category | | | |
|  | **Urban Non-Teaching** | 0.96 | 0.93 | 0.99 | 0.006 |
|  | **Urban Teaching** | 0.99 | 0.96 | 1.02 | 0.41 |
| **Hospital bed-size** | **Small** | Reference category | | | |
|  | **Medium** | 1.00 | 0.97 | 1.03 | 0.93 |
|  | **Large** | 1.02 | 0.99 | 1.04 | 0.31 |
| **Hospital region** | **Northeast** | Reference category | | | |
|  | **Midwest** | 0.98 | 0.95 | 1.00 | 0.08 |
|  | **South** | 1.02 | 1.00 | 1.04 | 0.07 |
|  | **West** | 0.94 | 0.92 | 0.97 | <0.001 |
| **Inter-hospital transfer** | | 0.86 | 0.84 | 0.88 | <0.001 |
| **Charlson Comorbidity Index** | **0-3** | Reference category | | | |
|  | **4-6** | 0.83 | 0.81 | 0.85 | <0.001 |
|  | **≥ 7** | 0.78 | 0.75 | 0.80 | <0.001 |
| **Acute organ failure** | **Hepatic** | 1.52 | 1.48 | 1.56 | <0.001 |
|  | **Hematologic** | 0.82 | 0.80 | 0.84 | <0.001 |
|  | **Neurologic** | 1.49 | 1.46 | 1.53 | <0.001 |
| **Severe sepsis** | | 1.01 | 0.98 | 1.04 | 0.57 |
| **Cardiac arrest** | | 2.59 | 2.53 | 2.64 | <0.001 |
| **Coronary angiography** | | 0.47 | 0.46 | 0.48 | <0.001 |
| **Percutaneous coronary intervention** | | 0.80 | 0.78 | 0.81 | <0.001 |
| **Invasive hemodynamic assessment** | | 0.99 | 0.97 | 1.01 | 0.34 |
| **Mechanical circulatory support** | | 1.11 | 1.09 | 1.13 | <0.001 |
| **Acute kidney injury** | | 1.39 | 1.37 | 1.42 | <0.001 |
| **Hemodialysis** | | 1.29 | 1.24 | 1.35 | <0.001 |

**Legend:** Hosmer and Lemeshow Test for goodness of fit: Chi-square 1019; *p*<0.001

**Abbreviations:** AMI: acute myocardial infarction; ARF: acute respiratory failure; CS: cardiogenic shock; MV: mechanical ventilation

**Table S3. Baseline characteristics of propensity-matched cohorts of AMI-CS**

| **Characteristic** | | **ARF with MV**  **(N = 9,240)** | **ARF without MV**  **(N = 9,240)** | ***P*** |
| --- | --- | --- | --- | --- |
| **Age group (years)** | **19-49** | 7.5 | 7.3 | 0.58 |
|  | **50-59** | 16.7 | 17.3 |  |
|  | **60-69** | 25.8 | 25.2 |  |
|  | **70-79** | 27.6 | 27.2 |  |
|  | **≥80** | 22.4 | 23.0 |  |
| **Female sex** | | 39.0 | 38.7 | 0.33 |
| **Race** | **White** | 58.9 | 60.1 | 0.07 |
|  | **Non-White** | 41.1 | 39.9 |  |
| **Hospital teaching**  **status and location** | **Rural** | 5.8 | 6.6 | 0.08 |
|  | **Urban non-teaching** | 41.3 | 40.2 |  |
|  | **Urban teaching** | 52.9 | 53.3 |  |
| **Hospital bed-size** | **Small** | 7.2 | 7.7 | 0.49 |
|  | **Medium** | 21.7 | 21.5 |  |
|  | **Large** | 71.1 | 70.8 |  |
| **Hospital region** | **Northeast** | 17.9 | 15.2 | 0.05 |
|  | **Midwest** | 22.7 | 22.5 |  |
|  | **South** | 36.3 | 45.6 |  |
|  | **West** | 23.1 | 16.7 |  |
| **Charlson Comorbidity Index** | **0-3** | 23.1 | 23.1 | 0.98 |
|  | **4-6** | 58.2 | 58.3 |  |
|  | **≥ 7** | 18.7 | 18.6 |  |
|  | **Renal** | 40.5 | 40.6 | 0.43 |
|  | **Hepatic** | 7.8 | 8.2 | 0.28 |
|  | **Hematological** | 12.7 | 12.6 | 0.43 |
|  | **Neurological** | 12.4 | 13.4 | 0.08 |
| **Cardiac arrest** | | 17.6 | 18.2 | 0.24 |
| **Coronary angiography** | | 77.1 | 75.9 | 0.05 |
| **Percutaneous coronary intervention** | | 57.5 | 56.6 | 0.22 |
| **Invasive hemodynamic assessment*** | | 21.7 | 22.3 | 0.33 |
| **Mechanical circulatory support** | | 57.8 | 57.0 | 0.25 |
| **Hemodialysis** | | 2.7 | 2.7 | 0.93 |

**Legend:** Represented as percentage; *right heart catheterization or pulmonary artery catheterization

**Abbreviations:** AMI: acute myocardial infarction; ARF: acute respiratory failure; CS: cardiogenic shock; MV: mechanical ventilation
