# Supplementary figures and images for: Acute respiratory failure and mechanical ventilation in cardiogenic shock complicating acute myocardial infarction in the USA, 2000–2014
Source: Ann Intensive Care. 2019 Aug 28;9:96. doi: 10.1186/s13613-019-0571-2 (PMC6713772; doi:10.1186/s13613-019-0571-2)

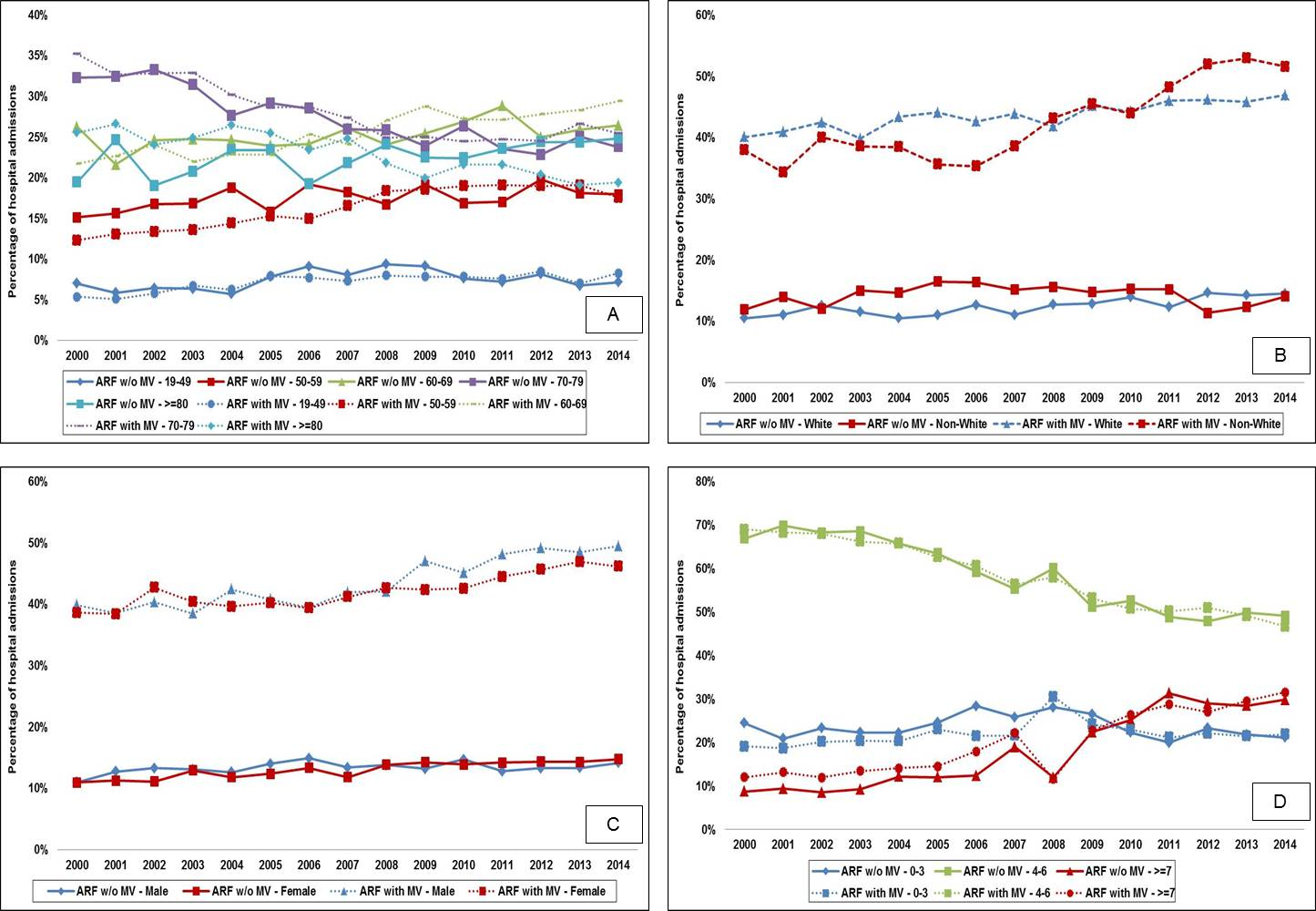

Supplement: Supplementary file 2 — Additional file 2: Figure S1. Trends of ARF and MV in AMI-CS stratified by demographic characteristics. Fifteen-year trends in acute respiratory failure (solid line) and mechanical ventilation (dashed line) in admission stratified by age groups (2A), race (2B), sex (2C) and Charlson comorbidity index groups (2D); all p < 0.001. AMI: acute myocardial infarction; ARF: acute respiratory failure; CS: cardiogenic shock; MV: mechanical ventilation. [file 13613_2019_571_MOESM2_ESM.tif]

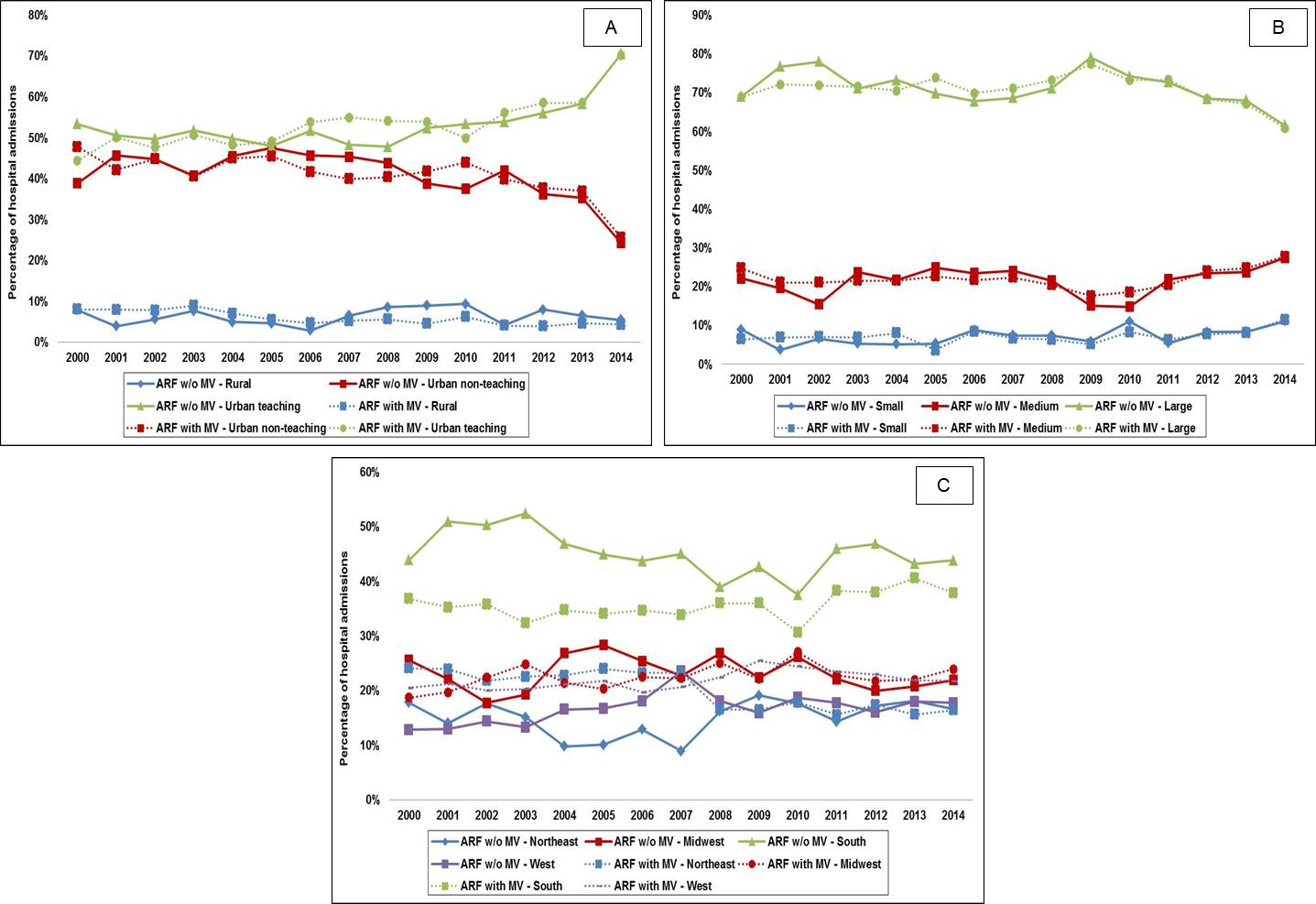

Supplement: Supplementary file 3 — Additional file 3: Figure S2. Trends of ARF and MV in AMI-CS stratified by hospital characteristics. Fifteen-year trends in acute respiratory failure (solid line) and mechanical ventilation (dashed line) in admission stratified by hospital location and teaching status (3A), hospital bed size (3B) and hospital region (3C); all p<0.001. AMI: acute myocardial infarction; ARF: acute respiratory failure; CS: cardiogenic shock; MV: mechanical ventilation. [file 13613_2019_571_MOESM3_ESM.tif]
